# Gene Context Analysis in the Integrated Microbial Genomes (IMG) Data Management System

**DOI:** 10.1371/journal.pone.0007979

**Published:** 2009-11-24

**Authors:** Konstantinos Mavromatis, Ken Chu, Natalia Ivanova, Sean D. Hooper, Victor M. Markowitz, Nikos C. Kyrpides

**Affiliations:** 1 Genome Biology Program, Department of Energy Joint Genome Institute, Walnut Creek, California, United States of America; 2 Biological Data Management and Technology Center, Lawrence Berkeley National Laboratory, Berkeley, California, United States of America; Technical University of Denmark, Denmark

## Abstract

Computational methods for determining the function of genes in newly sequenced genomes have been traditionally based on sequence similarity to genes whose function has been identified experimentally. Function prediction methods can be extended using gene context analysis approaches such as examining the conservation of chromosomal gene clusters, gene fusion events and co-occurrence profiles across genomes. Context analysis is based on the observation that functionally related genes are often having similar gene context and relies on the identification of such events across phylogenetically diverse collection of genomes. We have used the data management system of the Integrated Microbial Genomes (IMG) as the framework to implement and explore the power of gene context analysis methods because it provides one of the largest available genome integrations. Visualization and search tools to facilitate gene context analysis have been developed and applied across all publicly available archaeal and bacterial genomes in IMG. These computations are now maintained as part of IMG's regular genome content update cycle. IMG is available at: http://img.jgi.doe.gov.

## Introduction

Gene context analysis methods have proved to be valuable for genome structure and evolution studies as well as for protein function prediction [1], [2].

Computational methods for predicting the function of genes in newly sequenced genomes have been traditionally based on sequence similarity to genes whose function has been identified experimentally. Depending on the level of sequence similarity, functions predicted with such methods range from assignment to broad functional categories (e.g. aminotransferase protein family) to very precise function descriptions (e.g. alanine aminotransferase). Genes without similarity to functionally characterized genes are said to have unknown function and are often labeled as “hypothetical proteins”.

The rapid increase in the number of genome sequencing projects [3] has resulted in a growing number of so called “conserved hypothetical protein families” which denote predicted proteins conserved across a number of organisms, but without detectable sequence similarity to proteins of known function. In order to address this problem, new function prediction methods that do not depend on sequence similarity have been developed [1], [4], [5]. These methods are based on analyzing chromosomal gene clusters, gene fusion events, and occurrence profiles, and can be used jointly with similarity-based function prediction methods.

Chromosomal gene cluster analysis is based on the observation that functionally related genes are often collocated (i.e. in chromosomal proximity) forming transcriptional units (operons) in Bacteria and Archaea and operon-like gene arrangements in eukaryotes [1]. The fusion of two or more genes into a single gene often serves as evidence of their functional relationship [4]. Finally, gene co-occurrence profiles across organisms have also been shown to reflect functional relationships between genes. Proteins that function together in a pathway or structural complex evolve in a correlated fashion. During evolution, functionally linked proteins tend to be either preserved collectively, thus ensuring that the pathway or complex remains fully functional, or eliminated all together in a new species [2]. In addition to function prediction, context analysis can be used to delineate evolutionary patterns between organisms or to identify horizontal gene transfer events.

Context analysis relies on the availability of diverse sequence-based protein clusters across a large and phylogenetically rich collection of genomes. The Integrated Microbial Genomes (IMG) data management system is providing one of the largest available genome integrations and contains draft and complete genomes from all three domains of life [6]. IMG provides tools for examining genomes individually or jointly with other genomes in a comparative context.

We have extended the IMG analysis toolkit with gene context analysis visualization and search tools. These tools are applied to the conserved chromosomal gene clusters and fusion events that have been computationally identified across all archaeal and bacterial genomes in IMG. IMG is updated every four months with all the newly available public sequenced organisms, so its growing set of genomes allows gradual expansion of the coverage of the conserved chromosomal clusters and fusion events.

A small number of public systems such as PhydBac [7] and STRING [5] provide similar support for gene context type of analysis. This approach is different because it sets context analysis within IMG's richest comparative genome integration (over 1300 bacterial and archaeal genomes as of April 2009) with the new tools extending seamlessly IMG's analytical capabilities. Furthermore, this approach uniquely employs a combination of several gene clustering methods based on different protein family and domain characterizations, thus provide complementary, often more informative, views on functional relationships.

## Results

We have extended the Integrated Microbial Genomes (IMG) system with gene context analysis, visualization and search tools.

### Context Analysis Data

For IMG 2.8 (released in April 2009), the computation was carried out across 4.5 million genes distributed across 1343 archaeal and bacterial genomes and 974 plasmids and has resulted in the identification of 535,839 chromosomal cassettes and 265,935 fused genes. The number of conserved cassettes computed using COG, Pfam and IMG ortholog based clusters is shown on Table 1. As expected different types of protein families allow for different coverage of the protein space. The IMG orthologs cover almost 13% more genes than Pfam and 15% more than COG, but with a significant lower number of conserved chromosomal cassettes. This can be attributed to the nature of the IMG ortholog clustering that divides proteins with bidirectional best hits using the MCL algorithm, thus resulting in many more clusters of finer granularity. Pfam clusters on the other hand are more ubiquitous protein families spanning large number of genomes, and frequently found fused in many proteins. As a result the number of combinations of common Pfam neighborhoods across genomes is much larger as observed by the much higher number of Pfam conserved chromosomal cassettes.

**Table 1:** Statistics of conserved chromosomal cassettes in IMG 2.8.

	No of conserved cassettes	No of genes	% of total protein coding genes
COGs	8,653,081	3,114,028	68.71%
Pfam	29,858,323	3,222,379	71.10%
IMG ortholog	2,760,407	3,871,625	85.43%

Gene correlation coefficients (See Methods section) are also computed in order to provide metrics for the strength of relationships between pairs of protein clusters. Intuitively, protein clusters that appear frequently together in several genomes or in chromosomal neighborhoods or in fusion events are expected to have a stronger functional correlation. The maximum phylogenetic distance between organisms is used to adjust the correlation coefficients between multiple strains of the same species, where gene context conservation is more likely to reflect phylogenetic history rather than functional relationship between the genes.

### Context Analysis Tools and Viewers

**Figure 1:**
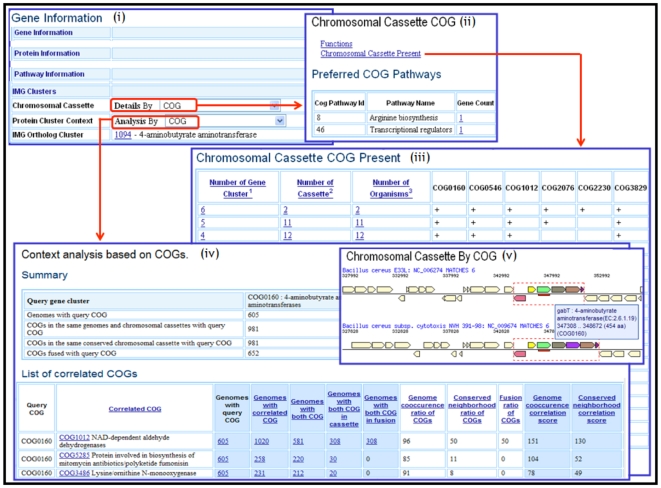
Gene specific chromosomal cassette details and viewers.

Information on a specific gene in IMG can be accessed through its Gene Details page, which includes information about the gene's protein family and domain characterization based on COGs, Pfams, TIGRfams, IMG ortholog clusters and enzymes. Context analysis tools are provided through the Gene Information section of Gene Details page, as illustrated in Figure 1(i). The details regarding the chromosomal cassette that includes the gene of interest, i.e. the query cassette, can be displayed through the Chromosomal Cassette page as illustrated in Figure 1(ii). This page provides information on the other genes, and their protein clusters (e.g., COGs), of the query cassette and the pathways they belong to, as well as information on other cassettes that share at least two protein clusters with the query cassette, as illustrated in Figure 1(iii). Next, Protein Cluster Context analysis allows accessing the functional correlations of the query protein cluster to all other clusters of the same type (i.e. COG, Pfam, IMG ortholog clusters), as illustrated in Figure 1(iv).

Starting with a query protein cluster A, the Context Analysis page contains a summary as illustrated in Figure 1(iv): for each cluster pair A, B the summary table lists:

- the number of genomes that contain individual genes associated with A, B, A and/or B, as well as cassettes or fusions that involve genes that are associated with A and B;
- the co-occurrence ratios for A and B, (see Methods section) i.e. the genome co-occurrence ratio of A and B, the conserved neighborhood ratio of A and B, the fusion ratio of A and B;
- the correlation scores for A and B i.e. the genome co-occurrence, conserved neighborhood and fusion correlation scores for A and B.

**Figure 2:**
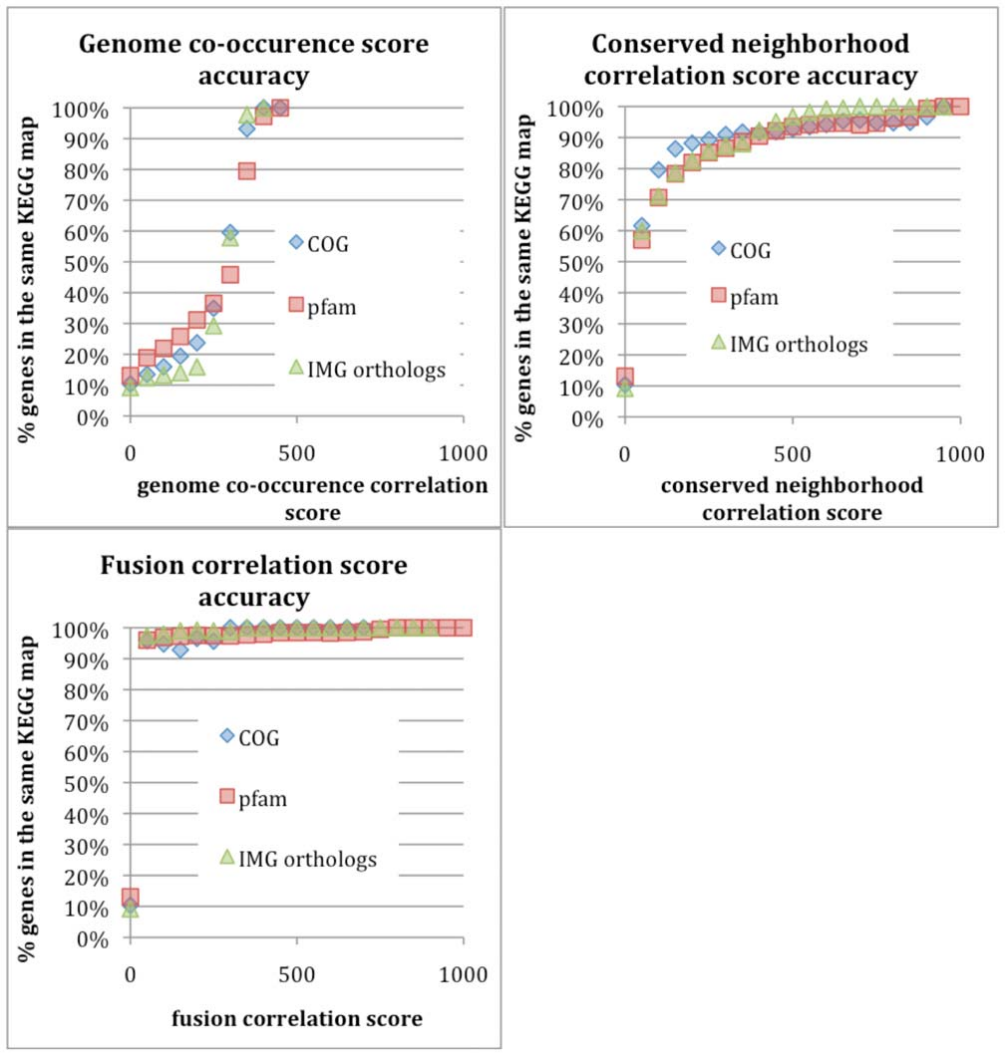
Percentage of protein families found in the same KEGG pathway at different correlation score levels.

The higher a correlation score is, the more likely a functional relationship between genes of the protein clusters is. The accuracy of gene correlation coefficients was evaluated using KEGG pathways. For every pair of studied genes that are associated with any KEGG map, we consider as true functionally related genes, genes that catalyze reactions on the same KEGG pathway. In Figure 2 we present the percentage of pairs of genes for any given conservation score that we expect to be related based on the previously mentioned criteria. Based on these data we can consider for example that gene pairs with conserved neighborhood correlation score above 200 have a probability of approximately 80% to be functionally related. Similarly any score above 50 can be used to predict functional relationship for fused gene pairs with a probability of more than 95%. For the genome co-occurrence correlation score the probability of identifying functionally related genes is lower than the other methods. Notably, in all cases, the number of gene pairs that exhibit high correlation scores and are functionally related with high probability, is small.

Chromosomal cassettes involving a specific gene can be examined using the Chromosomal Cassette Viewer, as illustrated in Figure 1(v). This viewer is available in the Evidence for Function Prediction section of Gene Details page, which also includes various other viewers for examining a gene. Chromosome cassettes can be viewed with genes labeled by their protein cluster (COG, Pfam, IMG orthologs) association. The query gene is denoted by a small red box under it, and for each chromosomal cassette, related cassettes in other genomes are also displayed. One can mouse over any gene to see its details. One can mouse over or click the red dotted line box surrounding a cassette to see the cassette details discussed above and illustrated in Figure 1(ii). Genes are colored by the protein cluster (e.g., COG) association, with genes that have no protein cluster or that are outside a cassette colored yellow.

**Figure 3:**
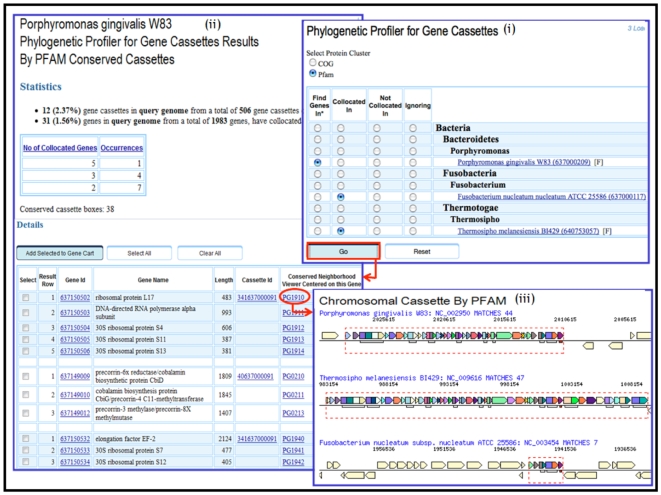
Phylogenetic Profiler for Gene Cassettes.

**Figure 4:**
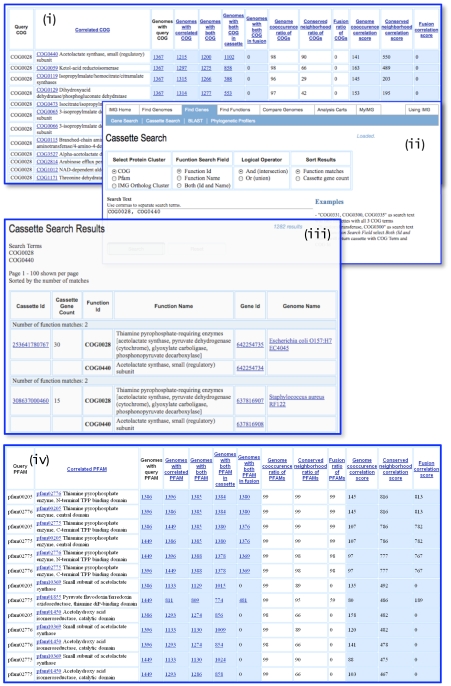
Separation of function of paralogous genes based on information of their chromosomal context.

Another context analysis approach supported in IMG involves the Phylogenetic Profiler for Gene Cassettes (found under the Find Genes tab), which allows selecting genes that are part of a gene cassette (i.e., are collocated on the chromosome) in a query genome and are part of related (conserved part of) gene cassettes in other genomes. First, a query genome needs to be selected by using the associated radio button in the “Find Genes In” column, as shown in Figure 3(i). Next, the type of protein cluster (COG, Pfam, IMG orthologs) used for correlating gene cassettes and the genomes for gene cassette comparisons are selected. The Phylogenetic Profiler for Gene Cassette Results displays a summary of the results, as shown in the top pane of Figure 3(ii), including a table with the first column listing the size of the groups of collocated genes in the query genome and the second column listing the number of such groups conserved across the other genomes involved in the selection. The Details part of the Phylogenetic Profiler for Gene Cassette Results consists of a table that displays groups of collocated genes in each chromosomal cassette (identified by the Cassette Id) in the query genome that satisfy the search criterion, as illustrated in the bottom pane of Figure 3(ii). Note that in each specific group of collocated genes in the query genome, individual genes may correspond to parts of multiple chromosomal cassettes in the other genomes involved in the profiler condition. Note also that the conserved part of a chromosomal cassette involving an individual gene in the query genome can be examined using the links provided in the “Conserved Neighborhood Viewer Centered on this Gene” column of results table, as shown in Figure 3(iii). Details of individual genes from the results list can be further examined by clicking on the associated “Gene ID”. Finally a search tool allowing searching for cassettes containing any combination of protein families is available [Figure 4 (ii)].

## Discussion

We have developed computational methods together with visualization and search tools that explore the power of gene context analysis within the comparative analysis framework of Integrated Microbial Genomes (IMG) data management system. Although similar methods and approaches have been reported by other groups in the past, this is the first time that gene context analysis is based on multiple protein clusters and applied to such a large number of genomes. Each of the three clustering methods has a different scope and allows different applications. For instance, Pfam is a clustering method based on local similarity, and can be used for the exploration of domain order conservation and shuffling across the phylogenetic space. On the other hand COGs, which group proteins with sequence similarity over the entire length, are more sensitive in detection of the overall protein relationships. IMG orthologs on the other hand are focused on computationally determined orthologs (BBH) and are limited to closely related organisms excluding paralogs from the same clusters. These differences are reflected in Table 1, where the number of Pfam based conserved cassettes is significantly higher than the counts produced by other two clustering methods due to the highly combinatorial nature of protein domains.

These new methods and tools in IMG, can be used to address important biological questions such as the identification of orthologs within large paralogous families and evolution of genome structure as well as function prediction of hypothetical protein families, or refinement of general function descriptions [8].

Accurate identification of equivalogs, i. e. orthologous genes that are presumed to have the same enzymatic or non-enzymatic function, is critically dependent on accurate identification of equivalogs, which may be difficult in large protein families that have undergone multiple events of duplication, deletion, neofunctionalization, and horizontal transfer. While identification of equivalogs within such families usually relies on phylogenetic analysis, gene context analysis using different types of protein clusters can be a valuable tool for the distinction between orthologous and paralogous genes.

Consider as an example a family of thiamine pyrophosphate-dependent enzymes described by COG0028, which includes proteins with various enzymatic activities, such as biosynthetic and catabolic acetolactate synthase, cytochrome-dependent pyruvate dehydrogenase and pyruvate oxidase, glyoxylate carboligase, phosphonopyruvate decarboxylase, and a number of other characterized and uncharacterized enzymes. This protein family has over 4600 representatives in IMG 2.8, with the number of representatives per genome ranging from 0 in the reduced genomes of obligate intracellular pathogens, such as *Mycoplasma spp.* to 17 found in the finished genome of highly versatile betaproteobacterium *Burkholderia xenovorans* LB400 [9], with the majority of bacterial and archaeal genomes having 2 or 3 members of this family. This family has very complex evolutionary history impeding accurate functional annotation of proteins belonging to COG0028; as a result many proteins are annotated merely as “thiamine pyrophosphate-dependent enzyme” or even as “hypothetical protein”.

Analysis of gene context of COG0028 using COG clusters and Protein Cluster Context analysis tool described above shows that members of this family are most often found in association with the regulatory subunit of acetolactate synthase and other enzymes from the pathway of branched chain amino acid biosynthesis, such as ketol-acid reductoisomerase, isopropylmalate synthase, etc. as shown in Figure 4(i), thus indicating that the majority of the representatives of this protein family have the function of the catalytic subunit of acetolactate synthase. A user can search for chromosomal cassettes that contain the genes assigned to the query COG and other COGs belonging to the same pathway [Figure 4(ii)] and find the genes that should be annotated as acetolactate synthase [Figure 4(iii)].

These results are further corroborated by gene context analysis of COG0028 members using Pfam clusters [Figure 4(iv)], which shows that the most highly correlated Pfams with PF02776, PF00205, and PF02775 corresponding to the N-terminal, central and C-terminal domains, respectively, are again Pfams representing the regulatory subunit of acetolactate synthase and ketol-acid reductoisomerase.

However, analysis of gene context using COGs and Pfams highlights different aspects of associations of this protein family and allows refinement of functional annotations for different subfamilies of COG0028. Pfam-based context emphasizes variation in domain structure and separates subfamilies with different domain combinations (e.g. PF01855 in combination with PF02776, PF00205 and PF02775 corresponds to pyruvate-ferredoxin oxidoreductase found in many anaerobes), while COG-based context assists in identification of proteins with different enzymatic activities and isozymes that are found in the vicinity of representatives of other COGs participating in the same pathway (e. g., catabolic acetolactate synthase participating in biosynthesis of acetoin and butanediol, which is associated with COG3527, alpha-acetolactate decarboxylase). In addition, some protein families are represented only as COGs, while others are represented only as Pfams; for instance, COG04032 [Figure 4(i)] has no equivalent Pfam, therefore proteins corresponding to a fusion of COG0028 and COG04032 can be detected only using COG-based context analysis.

Finally, due to a finer granularity of IMG clusters, analysis of context based on these clusters provides yet another tool for further refinement of annotation of proteins that cannot be distinguished through the COG- and Pfam-based gene context analysis. For example, functional descriptions of 3 members of COG0028 in *Burkholderia xenovorans* LB400 can be confirmed by IMG cluster-based context analysis: based on its association with the enzymes from branched-chain amino acid biosynthesis, the first protein represents the catalytic subunit of biosynthetic acetolactate synthase. The second protein is likely to be oxalyl-CoA decarboxylase, an enzyme catalyzing decarboxylation of oxalyl-CoA to formyl-CoA; this annotation is supported by association of this subfamily of COG0028 with formyl-coenzyme A transferase, which catalyzes the next reaction in the pathway. Function of the third protein is less obvious; however, analysis of proteins belonging to the IMG cluster 17497 (putative hydrolase) indicates that members of this cluster are likely to have phosphoenolpyruvate phosphomutase activity, which together with 2-aminoethylphosphonate-pyruvate transaminase is part of the pathway of aminophosphonate catabolism. Therefore, the third protein is likely to have the activity of phosphonopyruvate decarboxylase, an enzyme participating in the same pathway. Thus, gene context analysis approaches using different types of protein clusters are complementary and enhance our ability to perform accurate functional annotations and explore evolutionary histories of various protein families.

Cassette conservation may provide evidence of the evolution of the structure of the chromosome. Thus, cassettes that are conserved across large phylogenetic distances may indicate a common origin although in some cases horizontal transfer events involve the whole region. For example, IMG's Phylogenetic Profiler for Gene Cassettes can be used to search for collocated genes in the genomes of *P.gingivalis*, *F.nucleatum* and *T.melansiensis*, as shown in Figure 3(i). The result of this search is a list of genes located in 38 conserved chromosomal cassettes, including a cassette of ribosomal proteins as shown in Figure 3(ii). This chromosomal cassette exhibits remarkable conservation, as previously described [10]. Conversely, regions that are not conserved even across closely related organisms indicate hot spots of genome shuffling and gene loss. Thus, when comparing closely related organisms, the boundaries of conserved chromosomal cassettes suggest the location of such recombination hot spots.

Detailed exploration of the gene context data using these tools is available through the framework of IMG.

## Methods

### Context Based Gene Correlation

Gene correlations across genomes are identified using three alternative types of gene clustering based on: (i) COG cluster membership, (ii) Pfam assignments and (iii) ortholog (Bidirectional best hits) clusters implemented in IMG. While COG clusters group genes with overall sequence similarity and frequently similar functions, but not necessarily orthologous, Pfam based clusters group genes based on protein domains, while IMG ortholog clusters group together presumably orthologous genes, but typically in narrow phylogenetic groups. These alternative types of protein clusters result in potentially different gene contexts.

Context based correlation between genes is based on (a) collocation in the same chromosomal neighborhood, (b) fusion events, and (c) co-occurrence of genes across genomes.

A chromosomal neighborhood, also known as chromosomal cassette, is defined as a stretch of genes with intergenic distance smaller or equal to 300 base pairs [1]. The genes can be on the same or on different strands.

Chromosomal cassettes with a minimum size of two genes common in at least two different genomes are defined as conserved chromosomal cassettes. The identification of common genes across organisms is based on the three gene clustering methods mentioned above (i.e. participation in COG, Pfam and IMG ortholog gene clusters). For the purpose of assignment to COG and IMG clusters, if a protein has been identified previously as fused, it is assigned to the clusters of its components.

A fusion event involves the identification of a protein, which consists of two or more individual proteins co-occurring in at least two other organisms [11]. Fusion events are identified using pairwise similarities. Genes, such as transposases and integrases, pseudogenes, and genes from draft genomes are not considered as putative components in order to avoid false positives caused by gene fragmentation. The identification of fusions in IMG follows well established methods [4].

Gene co-occurrence refers to protein clusters that are found in the same group of genomes.

### Gene Correlation Metrics

Two correlation scores are defined as metrics of the relationship between a pair of gene clusters.

The first metric takes into account the phylogenetic distance between the organisms that contain the gene clusters. Given the above definition of a conserved chromosomal cassette we could observe a conserved pair of genomes between two strains of the same species, which could be the result of synteny and not functional relationship. The introduction of a term that includes the phylogenetic distance of the organism allows assigning more weight to the conserved chromosomal cassette found in more distant organisms. The phylogenetic distance between organisms is computed using a 16S rRNA based tree. The alignment of the 16S rRNA genes was extracted from the SILVA database [12]. The PHYLIP DNADIST program [13] was used to calculate the distance matrix between genes. For organisms for which a 16S rRNA gene has not been predicted (typically organisms at a draft sequencing stage), a 16S RNA gene from a similar organism (same species, or genus) was used.

For two gene clusters A and B (where both clusters represent COG, Pfam, or IMG ortholog clusters), their conserved neighborhood correlation score, or their fusion correlation score is computed according to the following formula:
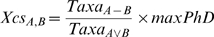

- Taxa A–B is the number of genomes where A and B belong to the same chromosomal cassette or are components of a fused gene,
- Taxa A V B is the number of genomes that have A or B, and
- maxPhD is the maximum phylogenetic distance between the organisms that have A and B, as part of the same cassette or fused gene.

Correspondingly their genome co-occurrence correlation score is computed according to the following formula:
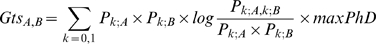

- P_k;A_ and P_k;B_ are the probabilities of a genome having (k = 1) or not having (k = 0) A and B, respectively;
- P_k;A,k;B_ is the probability of a genome having (k = 1) or not having (k = 0) both A and B as part of a cassette
- maxPhD is the maximum phylogenetic distance between the organisms that have A and B, potentially as part of the same cassette or fused gene.

The second metric is the ratio of the genomes that have both genes A and B (either in a conserved chromosomal cassette, or a fusion event, or in the same genome) divided by the minimum number of genomes that have either of the genes.

### Integrated Microbial Genomes (IMG) System

Conserved chromosomal cassettes and fusion events are computed across all publicly available archaeal and bacterial genomes in the IMG database. IMG is updated every four months with all the draft and complete genomes from all three domains of life that are available through RefSeq [14] including a large number of plasmids and viruses. The current version IMG 2.8 (April 2009) contains a total of 4,890 genomes consisting of 1,284 bacterial, 59 archaeal, 49 eukaryotic genomes, 2,524 viruses, and 974 plasmids that did not come from a specific genome sequencing project. For every genome in IMG, genes are associated with various protein clusters, including COG [15], Pfam [16] and IMG ortholog clusters, defined as bidirectional best hits of genes to other genomes clustered using the Markov Clustering (MCL) algorithm [17]. MCL was selected because it is a fast, non-supervised algorithm that allows the rapid clustering of data and has been extensively used for the clustering of biological data [18], [19].

Context analysis tools were developed in the framework of existing IMG data analysis tools. Genome data analysis in IMG consists of operations involving genomes, genes, and functions, which can be first selected and then explored individually [6]. Comparative analysis is provided through a number of tools that allow genomes to be compared in terms of gene content, functional capabilities, and sequence conservation. IMG's context analysis tools are further discussed in the next section.
